# Low Temperature Effect of Resistance Strain Gauge Based on Double-Layer Composite Film

**DOI:** 10.3390/mi17010114

**Published:** 2026-01-15

**Authors:** Mengqiu Li, Zhiyuan Hu, Fengming Ye, Jiaxiang Wang, Zhuoqing Yang

**Affiliations:** 1 National Key Laboratory of Advanced Micro and Nano Manufacture Technology, Shanghai Jiao Tong University, Shanghai 200240, China; li940619@sjtu.edu.cn (M.L.); hzy_sjtu@sjtu.edu.cn (Z.H.); fmye_21@sjtu.edu.cn (F.Y.); wangjiaxiang@sjtu.edu.cn (J.W.); 2 School of Integrated Circuits (School of Information Science and Electronic Engineering), Shanghai Jiao Tong University, Shanghai 200240, China

**Keywords:** strain gauges, temperature effect, double-layer composite film, ultra-near-zero

## Abstract

Strain gauges play a crucial role in numerous fields such as bridge and building structural health monitoring. However, traditional strain gauges generate spurious signals due to the temperature effect, which in turn affects their measurement accuracy. Herein, we propose a resistance strain gauge based on a double-layer composite film, which is characterized by an adjustable resistance temperature coefficient (TCR), an ultra-near-zero temperature effect, and good TCR repeatability. It is precisely through the combination of materials with positive and negative TCR, leveraging their opposing temperature resistance characteristics, that a low temperature effect has been achieved. Compared with the single-layer alloy-based strain gauge, the developed strain gauge based on double-layer composite film has greatly reduced sensitivity to temperature interference, and its TCR can be reduced to a ultra-near-zero value, approximately 0.8 ppm/°C, while the stability of TCR is excellent. In addition, the gauge factor of the strain gauge is 1.83, and it maintains excellent linearity. This work fully highlights the potential application value of the developed strain gauge in stress monitoring of bridges and building structures.

## 1. Introduction

In the construction and long-term maintenance of modern infrastructure, large-scale structures such as bridges and buildings are continuously subjected to multi-factor influences including dynamic loads, thermal cycling, and material degradation. These factors can induce micro-scale deformations and, over time, may escalate into significant structural damage [1,2,3]. Strain serves as a fundamental parameter for evaluating the stress state, and structural integrity of engineering components. By quantifying deformation, strain enables accurate assessment of structural safety and operational health. The primary function of a strain gauge is to transduce mechanical strain into a measurable change in electrical resistance. However, in practical operation, ambient temperature fluctuations independently induce resistance variations in the strain gauge, irrespective of mechanical strain. This temperature-induced interference, if not properly compensated, can be erroneously interpreted as a mechanical strain signal, leading to significant measurement inaccuracies [4,5]. The temperature coefficient of resistance (TCR) serves as a key metric for quantifying such temperature-induced interference. Moreover, in environments subject to substantial temperature fluctuations, strain gauges with inherently low TCR offer distinct advantages over systems relying on complex compensation circuits (e.g., half-bridge or full-bridge configurations) [6,7,8,9]. These low-TCR sensors can operate stably without external compensation circuitry. More importantly, in long-term monitoring applications—such as structural health monitoring of bridges and buildings—where ambient temperature varies cyclically with diurnal and seasonal changes, low-TCR strain gauges exhibit minimal resistance drift. This characteristic ensures consistency in long-term measurement data and helps prevent diagnostic errors arising from temperature-related artifacts [10].

Traditional rigid strain gauges utilize high-temperature-resistant alloys or ceramics—such as copper-nickel alloys, nickel-chromium alloys, and indium tin oxide—as strain-sensitive grid materials. These sensors offer a broad operating temperature range, with maximum service temperatures often exceeding 1000 °C. Nevertheless, their TCRs are typically 150 ppm/°C or higher [11,12,13], and only a limited number of studies have succeeded in reducing TCR to the order of tens of ppm/°C.

In this work, we propose a resistive strain gauge based on a double-layer composite film, which is characterized by ultra-near-zero TCR value, and good repeatability. Such excellent performance stems from the balanced effect of two materials with opposite TCR values. In the experiment, the resistive strain gauge based on the double-layer composite film is fabricated by Micro-Electro-Mechanical System (MEMS) technology, mainly including processes such as photolithographic patterning, sputtering, and lift-off. Theoretically, the optimal thickness ratio for combining two materials is derived through formula deduction. At this optimal thickness ratio, the TCR of the strain gauge is only 0.8 ppm/°C, with good cycle stability. Meanwhile, the resistance response to strain shows a highly linear change within the range of 400 µε. Therefore, the double-layer composite film-based strain gauge proposed in this work greatly weakens the interference of temperature on resistance, and fundamentally improves the measurement accuracy, environmental adaptability, and long-term stability of the strain gauge, enabling it to reliably capture real mechanical strain signals even under complex working conditions.

## 2. Materials and Methods

### 2.1. Materials

Al_2_O_3_ ceramic substrates were purchased from Shanghai Zhongke Shenguang Optoelectronic Industry Co., Ltd., China. Positive photoresist (model: AZ4620) was purchased from Suzhou Merck Electronic Materials Co., Ltd., Suzhou, China. Developer (model: AZ 400 K) was purchased from Merck Electronic Materials (Suzhou) Co., Ltd., China. CuNi and NiCr alloy targets were purchased from Zhongsheng Heng’an (Beijing) New Material Technology Co., Ltd., Beijing, China.

### 2.2. Fabrication of Double-Layer Composite Film-Based Strain Gauges

**Figure 1:**
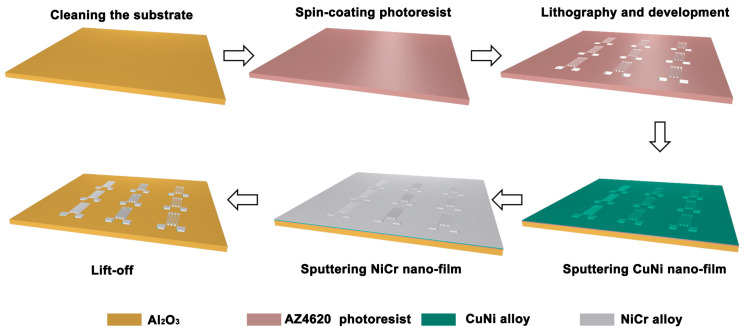
Fabrication process flow of double-layer composite film-based strain gauges.

As shown in Figure 1, the fabrication process of double-layer composite film-based strain gauges primarily involves MEMS technologies. First, the Al_2_O_3_ ceramic substrate is cleaned and dried. Subsequently, positive photoresist AZ4620 is spin-coated onto the substrate, followed by baking the photoresist at 90 °C. Afterward, photolithography and development are performed to achieve pattern formation. Next, CuNi alloy and NiCr alloy are sequentially deposited via magnetron sputtering. Upon completion of sputtering, the metal layers on the residual photoresist are stripped off. Finally, the sample is dried.

### 2.3. Characterization

The cross-sectional morphology of the thin films was observed using a field emission scanning electron microscope (Zeiss Sigma 300, Jena, Germany), with an EDS (Bruker Quantax XFlash SDD 6│30 Esprit 2.0, Berlin, Germany). The crystal structure and orientation of the double-layer composite films were analyzed by X-ray diffraction (XRD) using a D/Max-2500 X-ray diffractometer (Rigaku, Tokyo, Japan).

## 3. Results and Discussion

### 3.1. Characterization of Double-Layer Composite Films

**Figure 2:**
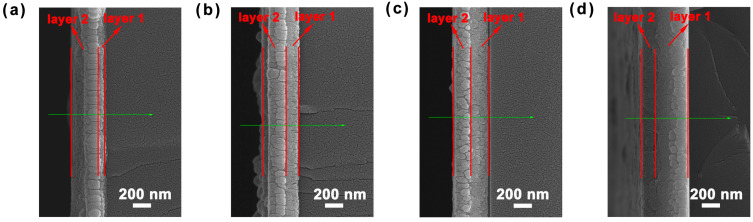
Cross-sectional SEM of double-layer composite films with different CuNi/NiCr thickness ratios: (**a**) d_0.26_, (**b**) d_0.56_, (**c**) d_0.94_, and (**d**) d_2.27_.

The cross-sectional SEM and EDS results of double-layer composite films with varying thickness ratios of CuNi to NiCr are presented in Figure 2. Herein, the notations d_0.25_, d_0.56_, d_0.94_, and d_2.27_ correspond to thickness ratios of two alloy films of 0.25:1, 0.56:1, 0.94:1, and 2.27:1, respectively, as shown in Figure 2a–d. As can be seen from the cross-sectional SEM, the film can be broadly divided into two layers: layer 1 corresponds to the CuNi alloy film, and layer 2 represents the NiCr alloy film. A distinct grain boundary between the CuNi and NiCr alloy films is clearly observed. The CuNi grains are small in size, with slight agglomeration occurring during the grain growth process. In contrast, the NiCr grains exhibit obvious columnar growth in the early stage of deposition, accompanied by more pronounced grain agglomeration. With the extension of CuNi alloy sputtering time, the thickness ratio of the CuNi to NiCr alloy films shows an increasing trend, which is consistent with the designed deposition time.

**Figure 3:**
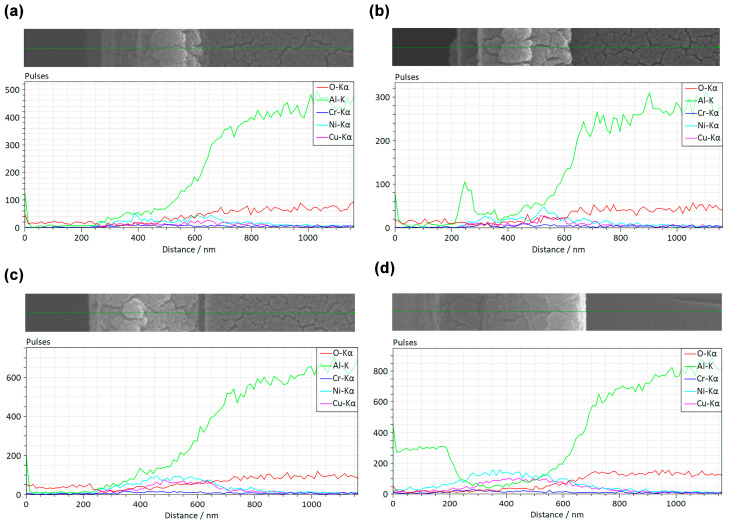
Locally magnified SEM and EDS of double-layer composite films: (**a**) d_0.26_, (**b**) d_0.56_, (**c**) d_0.94_, and (**d**) d_2.27_.

From the locally magnified SEM and EDS line scan in Figure 3b, it can be clearly observed that the substrate elements (Al, O) and the alloy elements of the lower-layer film (Cu, Ni) and the upper-layer film (Ni, Cr) exhibit trends such as stepwise variation, maintenance, decline, and stabilization with the change in scanning distance. Considering that Ni is an element common to both the upper and lower films, we separately investigated the distribution of chromium (Cr) and copper (Cu) content. It can be observed that, from the upper layer downward, the Cr content exhibits a slight increase followed by a decrease, while the Cu content also shows a distinct transition at the interface. These elemental distribution characteristics are consistent with the SEM and interface distribution, further confirming the well-defined layered structure of the composite films.

**Figure 4:**
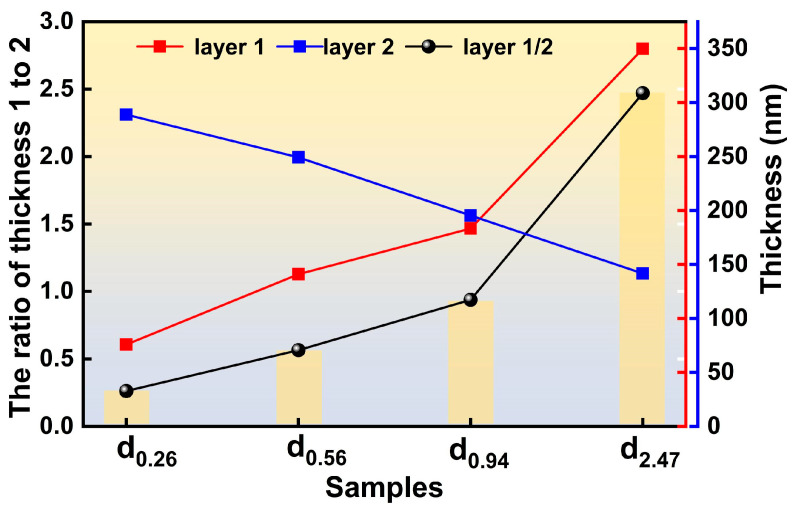
Thickness characterization of double-layer composite films.

Figure 4 presents the thicknesses of the CuNi and NiCr alloy films, and their respective thickness ratios in the double-layer composite structure. Considering the various influencing factors on film deposition rates during actual sputtering processes, the relative change in the thickness ratio (CuNi/NiCr) was used as the primary metric to evaluate film thickness variations. It can be clearly observed that the thickness ratios between the two alloy films differ significantly across the various samples. This variation in their thickness ratios provides a prerequisite for subsequent regulation of the temperature coefficient of resistance (TCR).

**Figure 5:**
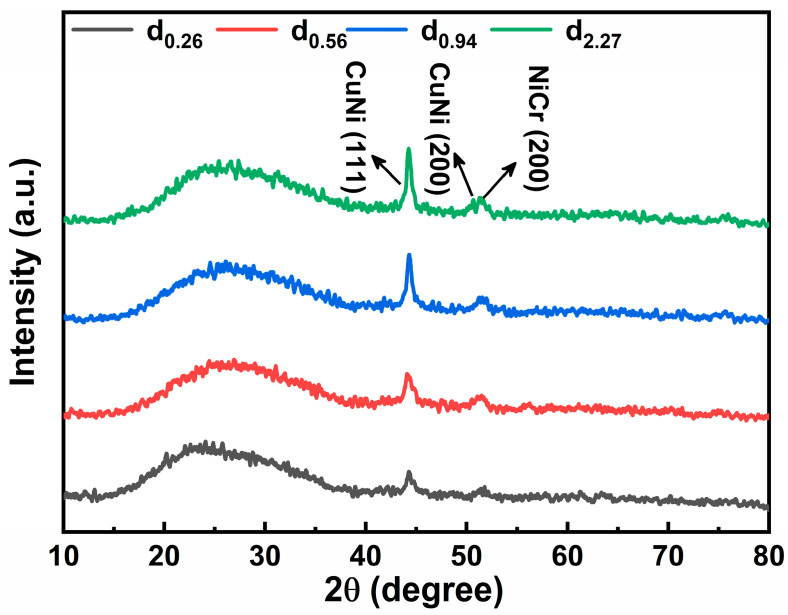
XRD patterns of double-layer composite films.

To quantitatively analyze the phase structure of the composite films under different thickness ratios, we performed an analysis of their XRD patterns. Considering that the Al_2_O_3_ substrate exhibits strong and sharp diffraction peaks that could mask those from the thin films, quartz glass substrates were used for the XRD characterization. As shown in Figure 5, the broad “halo” in the range of 15–35° originates from the amorphous glass substrate. It can be observed that as the CuNi/NiCr thickness ratio increases, the diffraction peak intensity near 44.3°, corresponding to the CuNi (111) plane, gradually increases. Additionally, the full width at half maximum (FWHM) of the peak near 51.5°, attributed to the NiCr (200) plane, progressively decreases, while a diffraction peak near 50.6°, corresponding to the CuNi (200) plane, emerges and becomes more distinct. These findings further indicate that in the double-layer composite film composed of CuNi and NiCr phases, the diffraction peaks associated with CuNi become increasingly dominant, suggesting a growing proportion of the CuNi phase relative to the NiCr phase.

### 3.2. Theoretical Derivation of Zero TCR for Composite Films

In alloy systems, the strengths of both lattice scattering (which contributes positively to TCR) and electron–electron interaction scattering (which contributes negatively) increase with rising temperature, thereby modulating the electron transport behavior within the material and ultimately determining its TCR characteristics. Furthermore, in disordered structures such as alloy thin films, defect- and grain boundary-related scattering centers—which exhibit negligible temperature dependence—can be categorized as static scattering. Although static scattering itself does not vary with temperature, its intensity can indirectly constrain the relative contributions of lattice scattering and electron–electron scattering, consequently influencing the magnitude of the TCR. The essence of near-zero TCR lies in the balance between the dynamic scattering mechanisms within the two alloys. For strain gauges based on double-layer films, the upper and lower layers are fabricated using NiCr alloy (Ni_80%_Cr_20%_) with a positive TCR and CuNi alloy (Cu_55%_Ni_45%_) with a negative TCR, respectively. According to the contribution of each layer to the total conductivity, the total conductivity of the double-layer film is obtained by the weighted sum of the conductivities of the two layers, where the weighting factors are their respective thickness ratios relative to the total film thickness. For double-layer films, the total TCR is determined by the contributions of each layer, which depend on their respective conductivities, TCR values, and thicknesses [14]. Based on the contribution of each layer to the total TCR, the relationship can be derived by Equation (1) as follows:

(1) $$\alpha_{total}=\frac{\sigma_{CuNi}TCR_{CuNi}d_{CuNi}+\sigma_{NiCr}TCR_{NiCr}d_{NiCr}}{\sigma_{CuNi}d_{CuNi}+\sigma_{NiCr}d_{NiCr}}\;\;$$

where *σ_total_*: total electrical conductivity of the double-layer film; *σ_CuNi_*: electrical conductivity of the CuNi alloy layer; *σ_NiCr_*: electrical conductivity of the NiCr alloy layer; *d_total_*: total thickness of the double-layer film, where *d_total_* = *d_CuNi_* + *d_NiCr_*; *d_CuNi_*: sputtered thickness of the CuNi alloy layer; and *d_NiCr_*: sputtered thickness of the NiCr alloy layer. *TCR_total_* denotes the total temperature coefficient of resistance of the double-layer film, *TCR_CuNi_* is the TCR value of the CuNi layer, *TCR_NiCr_* is the TCR value of the NiCr layer. It can be observed that the numerator of the equation is the sum of each layer’s conductivity, TCR value, and thickness, while the denominator is the sum of the products of each layer’s conductivity and thickness. To achieve a total TCR of zero (*TCR_total_* = 0) for the double-layer film, according to Equation (1), the numerator must be zero, as shown in Equation (2).

(2) $$\sigma_{CuNi}TCR_{CuNi}d_{CuNi}=-\sigma_{NiCr}TCR_{NiCr}d_{NiCr}\;\;$$

(3) $$\frac{d_{CuNi}}{d_{NiCr}}=-\frac{\sigma_{NiCr}TCR_{NiCr}}{\sigma_{CuNi}TCR_{CuNi}}\;$$

Given that electrical conductivity *σ* is the reciprocal of resistivity *ρ*, the zero TCR condition derived in Equation (3) can be equivalently expressed using resistivity as follows:

(4) $$\;\frac{d_{CuNi}}{d_{NiCr}}=-\frac{TCR_{CuNi}}{TCR_{NiCr}}*\frac{\rho_{NiCr}}{\rho_{CuNi}}$$

**Figure 6:**
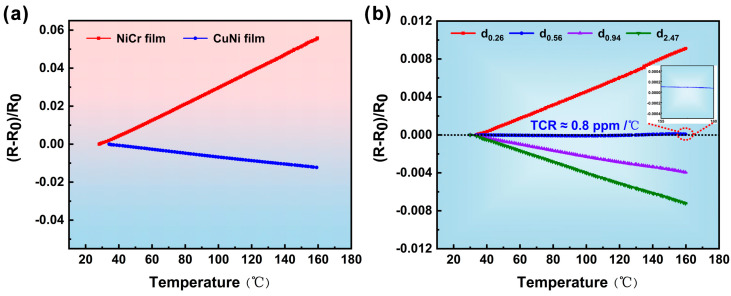
TCR characteristics of (**a**) single-layer CuNi and NiCr strain gauges, (**b**) double-layer composite film-based strain gauges with different CuNi/NiCr thickness ratios.

The theoretical TCR model discussed above primarily focuses on the analysis of temperature-sensitive dynamic scattering mechanisms and does not explicitly incorporate static scattering factors such as interfacial resistance, grain boundary effects, and thin-film size effects. While this model may exhibit minor deviations in quantitative predictions, it still captures the general trend and order-of-magnitude variation in TCR, thereby retaining significant theoretical reference value. Electrical resistivity and TCR measurements were conducted on single-layer CuNi and NiCr alloy films to determine their intrinsic electrical properties. The experimental results show that the CuNi alloy film exhibits a resistivity of 3.25 × 10^−4^ Ω·cm and a TCR value of −98 ppm/°C. In contrast, the NiCr alloy film demonstrates a higher resistivity of 7.36 × 10^−4^ Ω·cm and a positive TCR of 429 ppm/°C. These key electrical parameters are summarized in Figure 6a. The optimal theoretical thickness ratio of CuNi to NiCr alloy films for achieving a zero total TCR in the double-layer composite film-based strain gauge is calculated to be 0.53. This value is derived from the intrinsic electrical properties of the two alloys, confirming that temperature-induced resistance variations can be theoretically eliminated by balancing the negative TCR of CuNi with the positive TCR of NiCr through precise control of their thickness ratio. This theoretical calculation aligns with the aforementioned experimental result, in which the measured TCR approached approximately zero when the thickness ratio of the two alloys was 0.56. The consistency between the theoretical prediction and the experimental data confirms the validity of the model and the feasibility of achieving near-zero TCR through thickness ratio optimization.

### 3.3. TCR Characteristics of Composite Film Strain Gauges

The temperature coefficient of resistance (TCR) is a critical parameter that quantifies the sensitivity of a material’s resistance to temperature changes. For strain gauges, a low TCR value indicates minimal resistance variation induced by temperature fluctuations, which is essential for ensuring measurement accuracy and long-term stability in practical applications. The relationship between resistance and temperature for a material is mathematically described by Equation (5) [15]:

(5) $$\;TCR=\frac{∆R}{R_{0}∆T}$$

where Δ*R* represents the relative resistance change under the temperature change (Δ*T*), and *R*_0_ is the resistance corresponding to the initial temperature.

Considering that the actual operating temperatures of civil engineering structures (such as bridges and pavements) under extreme summer conditions typically remain below 90 °C, the upper limit of the temperature range used for TCR testing in this study has been extended to 160 °C. This range not only fully encompasses but substantially exceeds the temperature extremes encountered in practical applications, thereby ensuring the applicability and reliability of the research conclusions in real-world scenarios. As shown in Figure 6a, the TCR of the actually fabricated single-layer NiCr alloy film is approximately 429 ppm/°C, exhibiting a distinct positive TCR characteristic. In contrast, the single-layer CuNi alloy film demonstrates a distinct negative TCR characteristic with a measured TCR of approximately −98 ppm/°C. Notably, both films exhibit excellent linearity in their respective TCR curves, indicating stable temperature-dependent resistance responses within the tested temperature range. Furthermore, the opposite behavior of electrical resistance with temperature provides the fundamental possibility for tuning the overall TCR by adjusting the relative proportion of the two alloys.

To further investigate the effect of the thickness ratio of the double-layer composite film-based strain gauges, TCR tests were conducted on the four prepared samples (d_0.25_, d_0.56_, d_0.94_, d_2.27_). The test results are presented in Figure 6b. As observed, with the increasing thickness of the CuNi alloy film, the total TCR value of the double-layer composite film-based strain gauge gradually shifts from a positive value of 72.9 ppm/°C toward the negative direction. A critical transition occurs when the thickness ratio reaches 0.56: the TCR value reaches 0.8 ppm/°C, essentially achieving a true ultra-near-zero TCR contribution. This thickness ratio is fundamentally consistent with the value derived from the theoretical results, truly achieving minimal interference of temperature changes on the strain gauge’s resistance. As the thickness ratio further increases, the TCR shifts to a negative value of −30.3 ppm/°C; when the ratio reaches 2.47, the TCR is −56.3 ppm/°C, confirming that excessive CuNi thickness dominates the total TCR with its negative contribution. Compared with previous studies in Table 1 [16,17,18,19,20], the strain gauge based on the CuNi/NiCr double-layer thin film developed in this work exhibits an extremely low TCR, demonstrating significant potential for application in long-term strain monitoring under varying temperature conditions.

**Figure 7:**
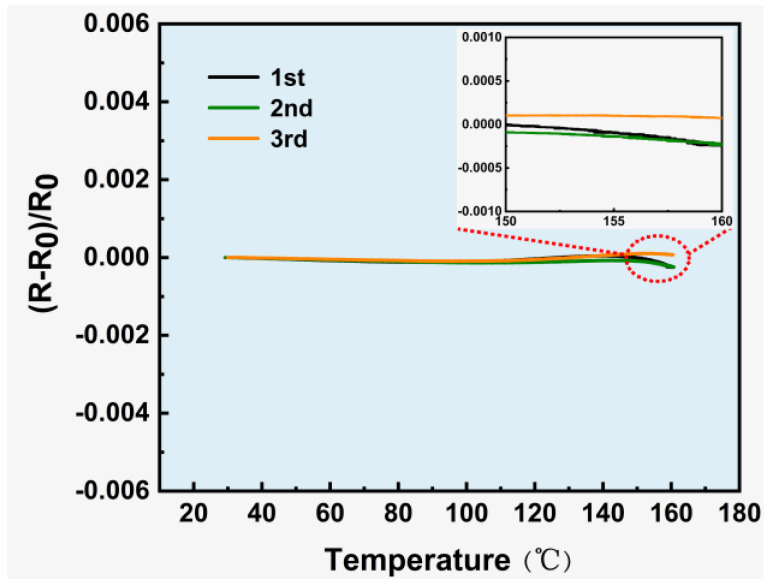
TCR cyclic stability of double-layer composite film-based strain gauges.

For the sample corresponding to the ultra-near-zero TCR thickness ratio, TCR repeatability tests were conducted, and the results are shown in Figure 7. The results of the three repeated tests are essentially consistent, with TCR values fluctuating within the range of ±1 ppm/°C. This high repeatability indicates stable temperature resistance characteristics of the composite structure under repeated thermal cycles. The double-layer composite film-based strain gauge fabricated in this work not only achieves ultra-near-zero temperature interference but also exhibits excellent repeatability, laying a solid foundation for its practical application in high-precision strain measurement scenarios with temperature fluctuations.

### 3.4. Strain Response of Composite Strain Gauges

The gauge factor (GF) of a strain gauge is a core parameter that quantifies its ability to sense deformation, directly determining the sensitivity of strain measurements. It describes the proportional relationship between the relative resistance change in the strain gauge and the applied mechanical strain, making it an indispensable key indicator in strain measurement applications. The relationship between resistance and strain for a strain gauge is mathematically expressed as follows [21]:

(6) $$\;GF=\frac{∆R}{R_{0}\varepsilon }\;\;\;\;$$

where ∆*R* represents the relative resistance change under the strain ε, and R_0_ is the resistance corresponding to initial temperature.

**Figure 8:**
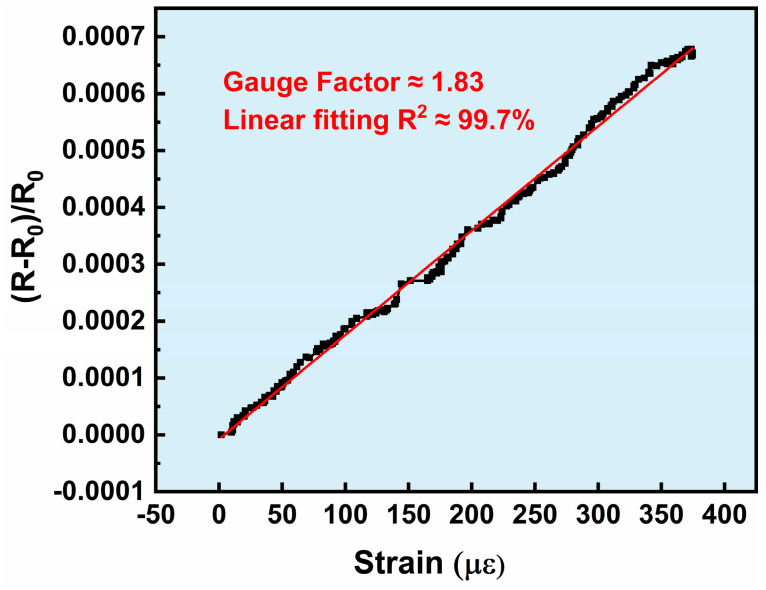
Strain response of double-layer composite film-based strain gauges.

The strain response of the double-layer composite film-based strain gauge is shown in Figure 8. It can be clearly observed that as the applied stress/strain increases, the resistance change rate also increases with good linearity. Within the range of up to 400 µε, the gauge factor of the strain gauge is 1.83, indicating that the strain gauge prepared in this work exhibits a stable resistance response to stress and strain. In this work, CuNi and NiCr were selected as the materials for the sensing grid, both of which are classic thin-film strain gauge materials. Strain gauges based on these materials typically exhibit a gauge factor of around 2. This specific value is predominantly determined by the intrinsic properties of the materials.

**Figure 9:**
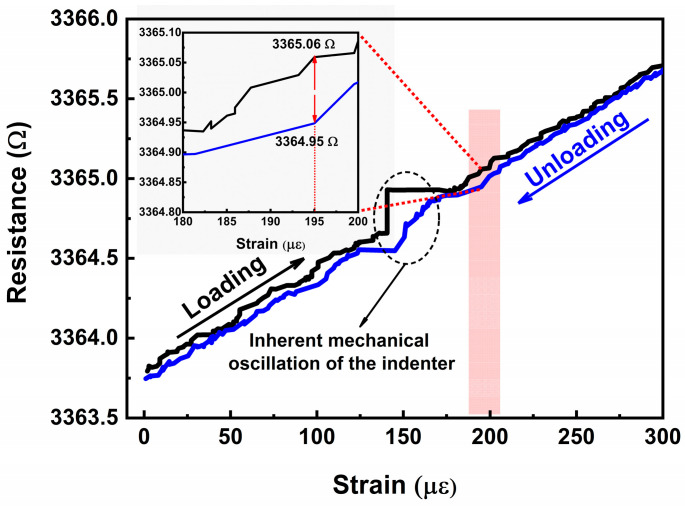
Hysteresis curves of the strain gauge during loading–unloading cycles with a 300 με full-range.

Figure 9 shows the hysteresis curves of the strain gauge during loading–unloading cycles with a 300 με full-range. It should be noted that the fluctuation observed around 150 με is caused by mechanical jitter of the indenter in the stress–strain testing system near that specific point, which represents a systemic artifact. Therefore, this region was excluded from the hysteresis calculation. The hysteresis error is defined as the maximum deviation between the resistance values recorded during loading and unloading at the same strain level.

(7) $$\;HysteresisError=\frac{{\left|R_{loading}-\right.\left.R_{unloading}\right|}_{max}}{R_{Fs}}\;\;\;\;$$

where *R_loading_* and *R_unloading_* denote the resistance during loading and unloading at a given strain, and ∣*R_loading_* − *R_unloading_∣_max_* is the maximum deviation between the loading and unloading resistance values at identical strain levels. R_FS_ refers to the full-scale resistance change.

As clearly observed in Figure 9, the unloading curve lies below the loading curve, with a full-scale resistance change (RFS) of 1.9 Ω. Furthermore, the enlarged inset reveals that at 195 µε, the Rloading and Runloading are 3365.06 Ω and 3364.95 Ω, respectively, where the difference reaches its maximum. The calculated hysteresis error is 0.0579.

**Figure 10:**
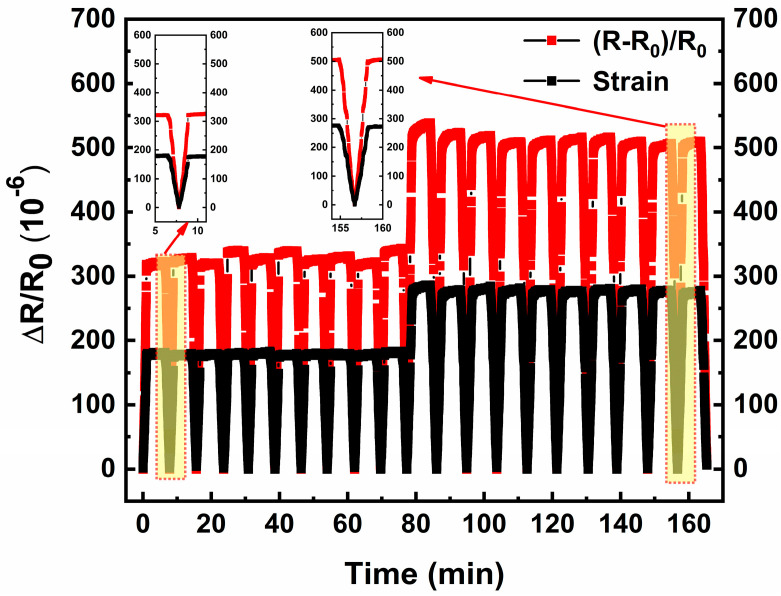
Cycling repeatability of the strain gauge.

Figure 10 shows the cycling repeatability of the strain gauge. As can be observed from the figure, the relative resistance change curve responds correspondingly to variations in the applied strain curve. Since the pressure was manually controlled via button operations, slight differences in the strain magnitude occurred between consecutive operations, resulting in minor variations in the peak values of the strain curves. Consequently, the resistance curves correspondingly reflect these fluctuations in the applied force. Moreover, compared with the response curve in the initial stage, the strain gauge maintains completely consistent resistance response to strain even after several hours.

## 4. Conclusions

In this work, we propose a double-layer composite film-based resistive strain gauge that achieves adjustable TCR, an ultra-near-zero TCR value, and excellent repeatability. This superior performance with low temperature interference is realized by leveraging the offsetting effect of two materials with opposite positive and negative TCR values. Based on theoretical formula derivation, combined with the relevant parameters of the two materials with opposite TCR values, the optimal thickness ratio of the double-layer composite film was calculated. The double-layer composite film-based resistive strain gauge in this work was fabricated using MEMS technology, mainly involving processes such as photolithography, lithography, sputtering, and lift-off. Experimentally, under this optimal thickness ratio, the strain gauge exhibits an extremely low TCR of only 0.8 ppm/°C, indicating minimal temperature interference. Meanwhile, within the range of 400 µε, the resistance response shows a highly linear variation.

Therefore, the double-layer composite film-based strain gauge proposed in this paper achieves the function of ultra-low near-zero TCR, significantly weakening the temperature-induced interference on resistance. This fundamentally improves the measurement accuracy, environmental adaptability, and long-term stability of the strain gauge, enabling it to reliably capture real mechanical strain signals even under complex working conditions.

## Figures and Tables

**Table 1:** TCR comparison of thin-film strain gauge.

Sensing Material	TCR (ppm/°C)	Ref.
ITO-Pt	−79	[16]
CuNi	7	[17]
PSZ/Pb_2_Ru_2_O_6_/TiB_2_	281	[18]
Karma	64.8	[19]
NiCrAl	−5	[20]
CuNi-NiCr	0.8	This work

## Data Availability

The data presented in this paper are available on request from the corresponding author. The data are not publicly available due to privacy restrictions.

## Abbreviations

The following abbreviations are used in this manuscript:

TCR	Temperature Coefficient of Resistance
MEMS	Micro-Electro-Mechanical Systems
GF	Gauge Factor
